# Web tools for large-scale 3D biological images and atlases

**DOI:** 10.1186/1471-2105-13-122

**Published:** 2012-06-07

**Authors:** Zsolt L Husz, Nicholas Burton, Bill Hill, Nestor Milyaev, Richard A Baldock

**Affiliations:** 1 MRC Human Genetics Unit, Institute of Genetics and Molecular Medicine, Western General Hospital, Crewe Road, UK; 2 Informatics, Edinburgh University, UK; 3 BAE Systems, Advanced Technology Centre, UK

## Abstract

**Background:**

Large-scale volumetric biomedical image data of three or more dimensions are a significant challenge for distributed browsing and visualisation. Many images now exceed 10GB which for most users is too large to handle in terms of computer RAM and network bandwidth. This is aggravated when users need to access tens or hundreds of such images from an archive. Here we solve the problem for 2D section views through archive data delivering compressed tiled images enabling users to browse through very-large volume data in the context of a standard web-browser. The system provides an interactive visualisation for grey-level and colour 3D images including multiple image layers and spatial-data overlay.

**Results:**

The standard Internet Imaging Protocol (IIP) has been extended to enable arbitrary 2D sectioning of 3D data as well a multi-layered images and indexed overlays. The extended protocol is termed IIP3D and we have implemented a matching server to deliver the protocol and a series of Ajax/Javascript client codes that will run in an Internet browser. We have tested the server software on a low-cost linux-based server for image volumes up to 135GB and 64 simultaneous users. The section views are delivered with response times independent of scale and orientation. The exemplar client provided multi-layer image views with user-controlled colour-filtering and overlays.

**Conclusions:**

Interactive browsing of arbitrary sections through large biomedical-image volumes is made possible by use of an extended internet protocol and efficient server-based image tiling. The tools open the possibility of enabling fast access to large image archives without the requirement of whole image download and client computers with very large memory configurations. The system was demonstrated using a range of medical and biomedical image data extending up to 135GB for a single image volume.

## Background

Multi-dimensional images are integral to biomedical research with many large scale imaging initiatives now in place to capture image data with genomic-scale coverage. These can be high resolution two- and three-dimensional (2D & 3D) and time varying images from the cellular through to whole organism level of resolution [1]. This data acquisition has been matched by the development of image archive and database systems to support the curation and query of the data [2,3]. Most of these systems will provide a number of visualisation options but in general these require the download of the full data-sets to be visualised using applications on the users’ workstation. In addition there are now a number of image databases that include a standard spatio-temporal reference framework onto which large volumes of data are spatially mapped to enable cross-comparison, query and analysis. Visualisation of these mapped data-sets could imply massive download of data beyond the local disk capacity and available bandwidth. In this context therefore the ability to access 3D image objects over a wide-area network such as the Internet is imperative.Recent work has demonstrated the clear advantages of tile-based image transmission and many *zoom-viewers* have been developed for example by Google Maps (http://maps.google.com), Zoomify (http://www.zoomify.com) and OpenZoom (http://www.openzoom.org). The key benefit is that the client application only requests enough data to display at the resolution required and therefore the data download scales in proportion to the view-window size which is in most cases constant. In addition the tile-based approach can benefit from data-compression, asynchronous download and the use of caching which makes very effective use of the network and provides a fast response for the user.

Some solutions involve the Internet Imaging Protocol (IIP)[4], which is an open protocol that provides fast tiled delivery of large images through a multi-resolution image representation. IIP has been used in tele-pathology and educational archives [5]. It allows a region of interest to be selected at any zoom level and provides efficient image transmission. An IIP server has been developed (part of IIPImage at SourceForge) as an open-source resource [6].

However, IIP (and all other related protocols) may only be used with 2D images and a similar presentation method for 3D objects did not exist. Therefore we have developed extensions to the IIP protocol, which we call *IIP3D*, and have implemented an open-source server to deliver this service based on the highly efficient *Woolz* image processing library [7,8]. In addition we have developed a number of browser-based applications for specific projects which we use to illustrate the capabilities of the system.

The importance of virtual slicing systems for remote access of images was previously noted [9] and the IIP protocol was identified as a suitable interface for independent client-server applications. However the availability and flexibility of these imaging systems was limited by the proprietary and costly nature of existing implementations. Current image servers, such as BrainMaps.org, are able to deliver 3D data although they provide only predefined 2D sections. Glatz-Krieger *et al.*[9] consider virtual slices only in the original focal planes of the biological material in the context of a 2D microscope slide. In this paper, we cut an *arbitrary* virtual section from the digitised 3D model. Such sectioning software exists, e.g. as commercial applications such as e.g. Amira [10] or open-source projects such as Slicer [11,12], ImageJ [13] or our own applications *MAPAINT*[14] and the Java atlas viewer, JAtlasViewer viewers [15]. There are also online Java applications such as NeuroTerrain [16]. Java is often selected for compatibility and platform independence, but sometimes falls short of this in practice.

Tile based image delivery, that transmits the target as smaller image blocks, is known from commercial web applications such as Google maps. This runs in any web browser and does not require additional software or an applet. We have developed a server based on the open IIP protocol that can deliver arbitrary sections through large scale 3D image data providing a very fast and efficient display that can be accessed with standard browsers. The Visible Human project has generated several Internet based image servers and clients. The EPFL server [17] is the most similar to ours. It is a high throughput parallelised sectioning server using a FastCGI (FCGI) web interface. However it does not allow tiled requests and has a proprietary protocol. In addition, for the delivery of section data, the EPFL server requires a high performance cluster. In contrast, the IIP3D server will run on standard Linux-based servers, is open source and has been tested under Linux, Mac OS X and Windows.

To supplement the viewing of arbitrary sections through 3D image data we have developed clients which can visualise any number of image layers including indexed graphical overlays. Many “atlas” views of data include the display of graphically defined regions that correspond to a segmentation of the image space. Typically these atlas regions are constrained in number (usually 255 in the context of an 8-bit index image) and constrained spatially not to allow overlaps. In contrast we have developed an index image that can have an arbitrary number of regions with any combination of overlapping regions and these are provided as an indexed overlay with complete control over the colour and opacity of the individual regions.

In summary, we have extended an open protocol and associated tools which allow fast presentation of 3D volumetric data as arbitrary cut sections delivered at the level of detail and localisation requested, in the form of compressed tiled images. This system is open source, generic and integrable with other functionalities. This extended protocol is *IIP3D* and in the next section we present the implementation, followed by details of the server and client software. We present results of performance testing with multiple simultaneous access and show how the client software can be utilised for a number of atlas-based and large scale volume image delivery.

## Implementation

### Woolz image representation

Core to this work is the image processing library known as *Woolz*[7]. Woolz is unusual in that it has separate data structures for the spatial domain of an image object and the image values, which can be 8, 16, 32 bit grey values, RGB *α* or float/double. The domain of an image object is simply the region (arbitrary point-set) of 2-D or 3-D space over which the image is defined. This form of record is particularly appropriate for biomedical images, as it allows a compact representation of images through not storing background values outside of the foreground object of interest and it encodes arbitrary regions of space (such as anatomical and gene expression domains) without the need for image values. Internally Woolz represents a 2D domain as a number of line segments or *intervals* for each line for which there is image information. In this way an arbitrary region of 2D space can be defined in a very compact form and binary operations can be reduced to a series of interval comparisons which are very efficient for binary-set, morphological and labelling operations. A 3D domain is simply a planewise stack of 2D domains and null entries for planes which are entirely outside of the domain.Each Woolz object has its own coordinate system defined with respect to a global origin of all objects and three coordinates *kl* and *p* for the columns, lines and planes respectively. The objects have uniform sampling and real world coordinates are achieved through a voxel size (spatial sampling rate for the voxels) which is stored in the domain. Because objects can be located at any coordinate location, including negative values, the domain and value table are defined relative to the overall image bounding box which allows for very simple re-location of image data. Note conversion from Woolz to more standard rectangular domain based image formats may lose this information. Converters exist to transform Woolz image objects to a wide range of other formats including NIfTI, VTK, Amira and standard tiff-stacks.

#### Large Woolz objects

A recent extension of Woolz allows operations on large objects (e.g. up to terabyte volumes) that do not fit in system memory or would take many minutes to load from disk. It uses memory-mapped files, which reside on the external memory and are loaded on-the-fly block by block as the system requires it. 3D memory mapped objects are represented as a domain and grey value pair, however the grey values are stored in fixed size cuboidal blocks, accessed through a lookup table. The image value subdivision into cubes is similar to other solutions [16,17] but uses memory mapping and file-system block alignment. These block sizes are integer multiples of the file system blocks enabling very efficient read and write operations. With solid-state disks the I/O rate is similar to data that resides on the main memory.

#### Cutting arbitrary sections

**Figure 1:**
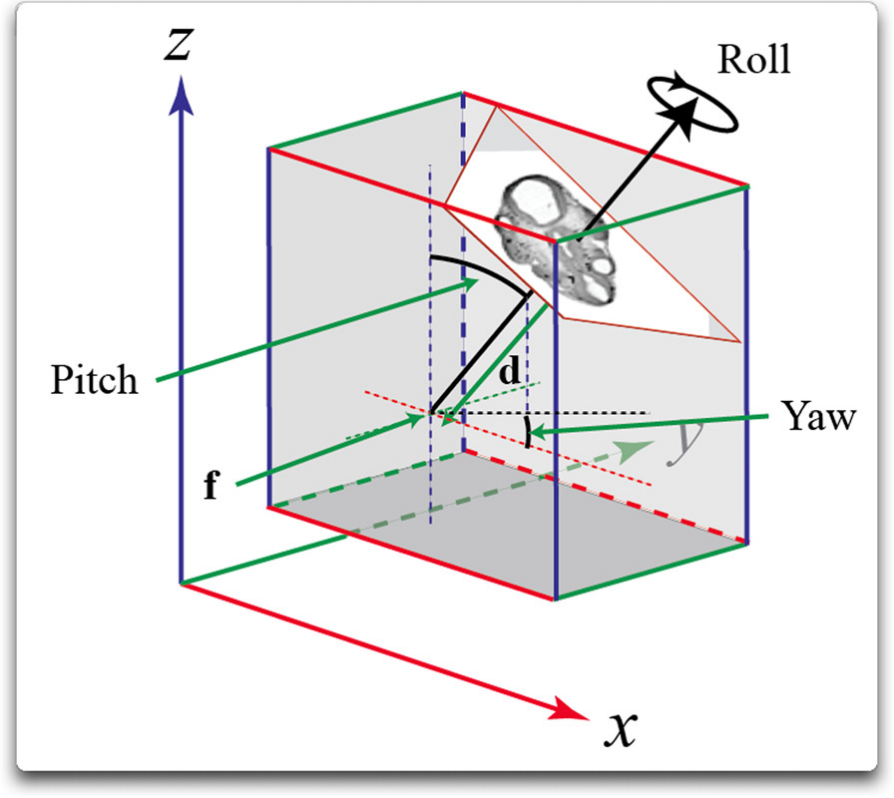
**Sectioning plane definition.** The viewing plans plane is at distance *d* from the fixed point **f** and perpendicular to the direction defined by angles *pitch* and *yaw*. The angle *roll* defines the orientation of section image when projected onto the view window which in our case is the user’s screen.

We specify an arbitrary section through the data volume as a plane perpendicular to the *line-of-sight*. This line is determined by three angles of rotation, two to establish the line direction and the third the orientation around that line. These correspond to pitch, yaw and roll and can be defined in terms of the standard Euler angles [18]. The rotation centre is the “fixed point” and the section plane is set by the distance along the view direction. The details of the transform are published [19], are given in the MAPaint technical report [14] and are illustrated in Figure 1. It is defined in terms of the fixed point **f**, angles *pitchyaw* and *rollscale* and distance *d*. The viewing transform can be represented as an affine transform where the section plane is the plane of constant *z*^*″*^ = *d*. By representing the transform in this fashion is straightforward to include any further re-scaling arising from non-isotropic voxel sizes. These underlying coordinate transformation methods have been extensively used for developing the eMouseAtlas models and gene-expression database and are part of the essential data captured as part of each submission. Convenient navigation with fewer free parameters is provided by a number of standard viewing modes defined in Table 1.

**Table 1:** Viewing modes

Mode	Purpose
Statue	The viewer is fixed and the cut section is viewed by rotating about the line of intersection with the original *z*-plane. With non-zero pitch the effect of changing yaw will result in the section appearing to rotate.
Up-is-Up	A pre-defined or user-defined vector **up** is projected onto the cut section which is then rotated so that it will be parallel to the vertical axis on the user’s screen.
Fixed line	The fixed point used to define the centre of rotation can be coupled with a second fixed point to define a fixed *line* leaving one d.o.f. of rotation around that line.
Absolute	Unconstrained viewing mode where all angles must be defined explicitly.

### IIP3D server

The IIP3D server is C++ software, based on a GNU GPL implementation of the IIP server by Pillay and Pitzalis [6]. This is a FastCGI (FCGI) web server module that is managed by the web server (e.g. Apache) or as a standalone application and is connected by the web server as requests arrive.

The full technical detail of the Woolz extensions of the IIP was provided earlier[20,21], here we present a limited set of IIP3D commands.

#### IIP3D request

The IIP3D server requests are parametrised HTTP requests and typically have four components: **Server address:** identifies the IIP3D server. Here we use the HTTP protocol and therefore the address is the URL of the FCGI application e.g. . **Resource Specifier:** the second component of a request defines the image resource from which tiles are requested. This for pyramidal TIFFs is , while for 3D Woolz objects is the command. **View parameters:** specifies the *parameters* of the sectioning plane (e.g. angles, distance) and the image return (e.g. format and compression) as well as setting for overlay colours and opacities. **Information request:** defines the specific information required from the object and sectioning plane set. This can be a tile, a full section or parameters of the object or sectioning plane (e.g. object dimensions, sectioning plane size, grey value or distance of a point from the sectioning plane, *etc.*).

The supported image request commands are similar to the IIP[4]: returns the full image (jpeg or png); while and the jpeg-, png-compressed and uncompressed tiles. IIP3D also supports PNG format with lossless compression and alpha channel, both crucial for painted domain or textual overlays.

3D browsing is essentially the selection of a section by setting the various parameters defined by the , , , and commands. The IIP3D protocol also supports the viewing modes from Table 1 which given *pitch* and *roll* will define *yaw*.

In addition the IIP3D server can provide information on the 3D object, with the available queries listed in Table 2. These include information about the 3D image volume, coordinate transforms between the displayed tiles and the original 3D image, image value and true voxel sizes.

**Table 2:** IIP3D object request extension summary

Object	Purpose
IIP-server	Identify if IIP3D server is running
Max-size	The size of the section
Tile-size	The size of a tile
Wlz-3d-bounding-box	The first and last plane, line and column number of the object
Wlz-coordinate-3D	The 3D coordinates defined in 2D by the command
Wlz-distance-range	The range of the sectioning plane distance
Wlz-foreground-objects	The index value of a compound object that has foreground pixel at a point specified either the or the command
Wlz-grey-value	The grey or RGB value of a point specified either the or the command
Wlz-sectioning-angles	The pitch, yaw and roll angles of of the sectioning plane
Wlz-transformed-coordinate-3d	Returns the the display coordinates of the projected 3D point defined by PAB and its distance from the sectioning plane
Wlz-true-voxel-size	The voxel size of the object
Wlz-volume	The volume of the object

#### Image Overlays

In a number of atlases, the histological image may have overlay layers presenting for example anatomical domains or gene expression patterns. These are analogous to satellite images overlaid with street map or traffic information. IIP3D provides this functionality with the select () command and uses the compound Woolz object format to capture the set of domains. This is an array of independent Woolz objects stored in a single file and each domain can be accessed by selecting the corresponding index. Any number of such indexed domains are allowed and there are no spatial restrictions, the domains can overlap within the context of the viewed section and will be displayed as a set of overlapping colours.

The most general syntax of is where is the selected objects index, while , , , are red, blue, green and alpha channels specifiers.The RGB components paint with the chosen colour the selected domain, or for grey/colour objects filter the image selecting only the desired colour components. Simplified syntax allow 1, 2 and 4 parameters with ; and , with 255 as the default for the omitted parameters.

Grey level or domain components are either independently selectable or stacked in an arbitrary order to generate grey level images, semi-transparent mask images, or a combination. A sequence of arbitrary number of commands specifies the composition order with the alpha channel information to determine layer visibility.

The requests the index or image value at query point allowing augmented display (e.g. name & description of a domain) in the viewer application. Uniquely the compound object based representation allows multiple overlaying domains to be displayed and queried which implies that the index query will in general return a list.

#### Caching

Faster image delivery is supported by three caching levels. As in the original IIP server, the IIP3D server caches the requested tiles. If multiple users access the same data, or it returns to a previously visualised region then the tiles are not recomputed but fetched directly from the cache. 3D images are often large, and the disk-read time is a significant overhead. Therefore woolz image objects once read are cached for further operations. However, for very large, memory mapped objects only the domain is cached, and not the values. Sections transforms which provide a fast look-up-table encoding of the affine transform are also cached.

#### Extreme data sizes

Biomedical images may easily be many GB in size, for example the Visible Human image is about 17.5 GB and data sets for electron-microscopy data now reach many terabytes [22]. Most current desktop workstations do not have sufficient memory to load such images and are incapable of being upgraded to be able to do so. Even when a workstation is capable of reading such a large object into memory the time taken to transfer it from disk is usually inconvenient; with a disk based file system capable of transferring data at 40MB/s it would take at least 7.4 minutes to read the Visible Human image into memory.

If the image is part of a resource then downloading significant data is very slow and copying multi-terabyte volumes infeasible. In most cases however, only a small subset of the image data is needed for visualisation since the typical user will be restricted by screen size. For this case we have extended the IIP3D server to use the memory-mapped option for very large woolz images enabling access to images of tens or hundreds of gigabytes.

#### Supported Image Formats

The woolz imaging system can convert from a range of 2D and 3D formats and are therefore directly supported by the IIP3D server. Supported formats include: Amira lattice, Stanford density, gif, Analyze, IPLab, Jpeg, BioRad Confocal, PNM, raw image data, Tiff, Nifti and vtk. To get the benefit of memory-mapped option for very large volumes and the domain overlays with a global coordinate system, it is necessary to convert to woolz format data.

### IIP3D Viewer

The IIP3D Viewer is a configurable web application which displays tiled images requested from a IIP3D Server. It was initially based on the viewer of Pillay [23] but has now been substantially developed and re-organised. The IIP3D viewer uses a number of web technologies including: HTML/HTTP, PHP/JSP, CSS, JavaScript, AJAX (with JSON).

A JSON configuration file identifies various system parameters including the location of the image and the IIP3D server. It also defines the parameters of all the layers associated with an image, including name, location of data and display order. If a tree control is to be used (e.g. for selecting overlay data), the structure and content of the tree is defined in a configuration file.

**Figure 2:**
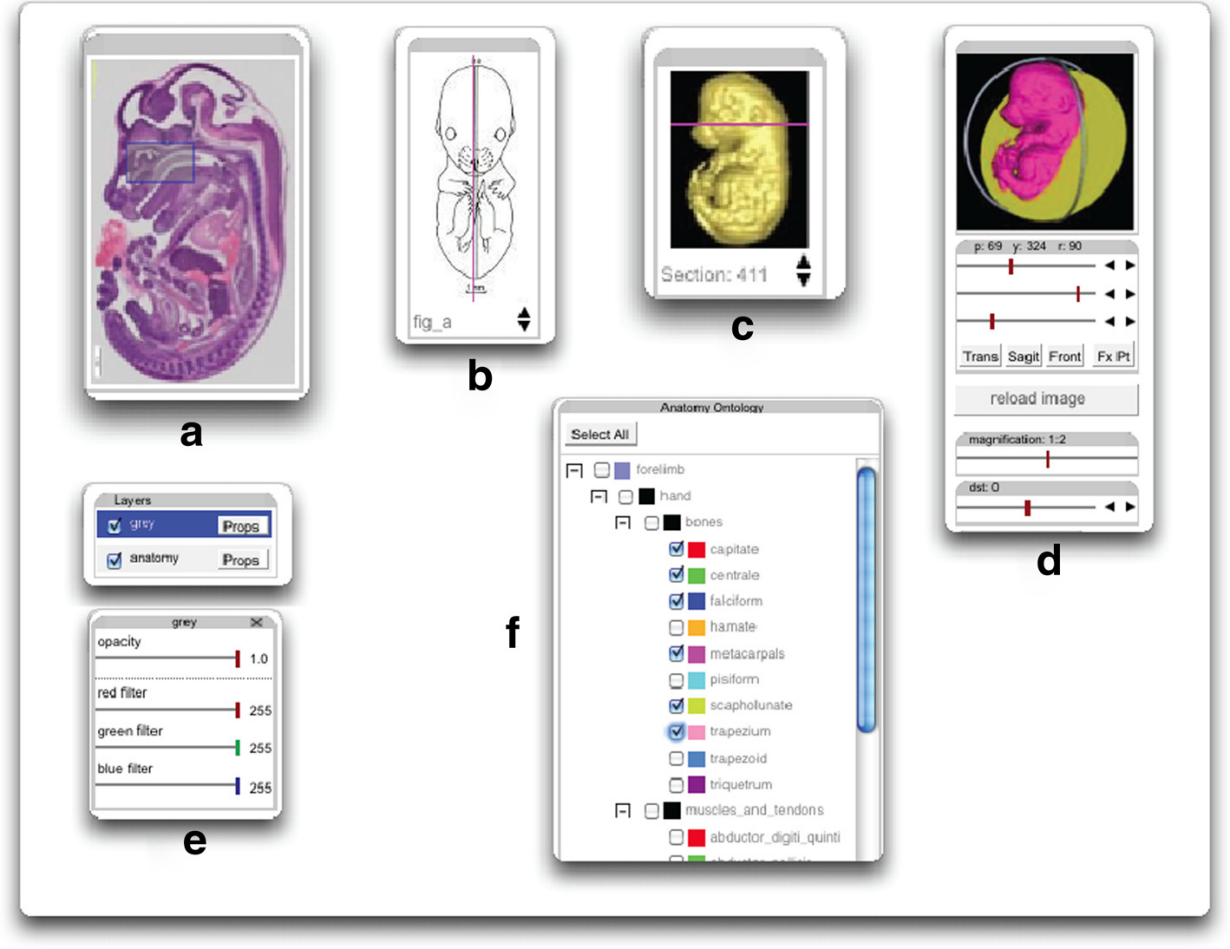
**Example controls for the IIP3D viewer: a - image locator, the blue rectangle indicates the currently visible region and can be dragged to any required location within the full image; b & c - image selection, the red line is dragged to select a new section image; d - 3D section controls with the angle feedback at the top and specific sliders for pitch, yaw, roll, scale and distance.e** - layer controls selecting visibility, opacity and if needed a colour filter to distinguish multiple grey-level image overlays; **f** - graphical overlay controls in this case for anatomy regions with the option to control visibility and colour.

The central area of the Viewer displays the visible region of an image. Tools for user interaction with the image will typically also be included. The choice of tools and their respective position within the web page is configurable by the web page developer via another JSON file. Tools, see Figure 2 for a selection, are available which allow for: changing the visible region of an image; changing the zoom level (resolution) of the displayed tiles; toggling the visibility of each layer on/off; changing the opacity of each layer; changing the colour filtering to be applied to grey-level layers (useful for comparing the registration of two overlaid grey-level images); for 3D Wlz images changing the sectioning plane (pitch, yaw and roll angles and distance of the sectioning plane from a fixed point, respectively for stacks of 2D images: changing the selected section within the stack (like choosing a card in a deck of playing cards).

If overlaid data is available and has been defined in the configuration file, it is possible to generate feed-back information by use of a ’mouse-down’ action over the image. This can be used, for example, to identify structure names within an image. With a small amount of JavaScript coding it is possible for a web-page developer to add their own custom tools.

The browser tries to be efficient in terms of transmission bandwidth and client memory by: requesting tiles only for the visible region of an image; requesting tiles only at the current resolution; for a scrolled/panned object, only new tiles are requested; layers which are not visible are not loaded.

**Figure 3:**
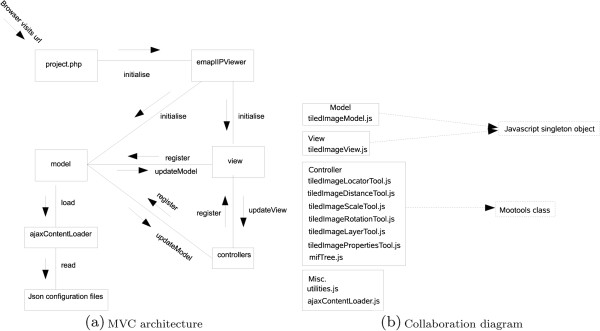
**IIP3D Viewer design is based on the MVC design pattern: (a) Sequence of a web page request is server by a sequence of php and JavaScript pages that implement the MVC.** Asynchronous tile image load is provided by AJAX; (**b**) Controllers provides control through the UI of Mootols library. The model encodes current viewing parameters set by the controllers and visualised by the tileImageView.

Figure 3(a) describes the basic interaction of the components of the IIP3D Viewer. As far as possible, the *Model View and Controller*(MVC) Figure 3(b) aspects of the viewer have been separated adopting a standard MVC design pattern.

### IIP3D Proxy

In the case where a large image archive it to made accessible via HTTP requests it is often not desirable to have the web-server also act as the image server. In addition with a large archive or high access rates the requests may need to be delegated to a number of servers. To provide a single point of access and enable this delegation to image servers separated from the Internet by a firewall, we have developed IIP3DProxy, an FCGI application, which filters requests and forwards them to a set of IIP3D servers. The communication conforms to the FCGI protocol. Though it was designated to work for IIP and Woolz requests, it is generic and can route any FCGI request, hence it allows chaining of multiple proxies.

**Figure 4:**
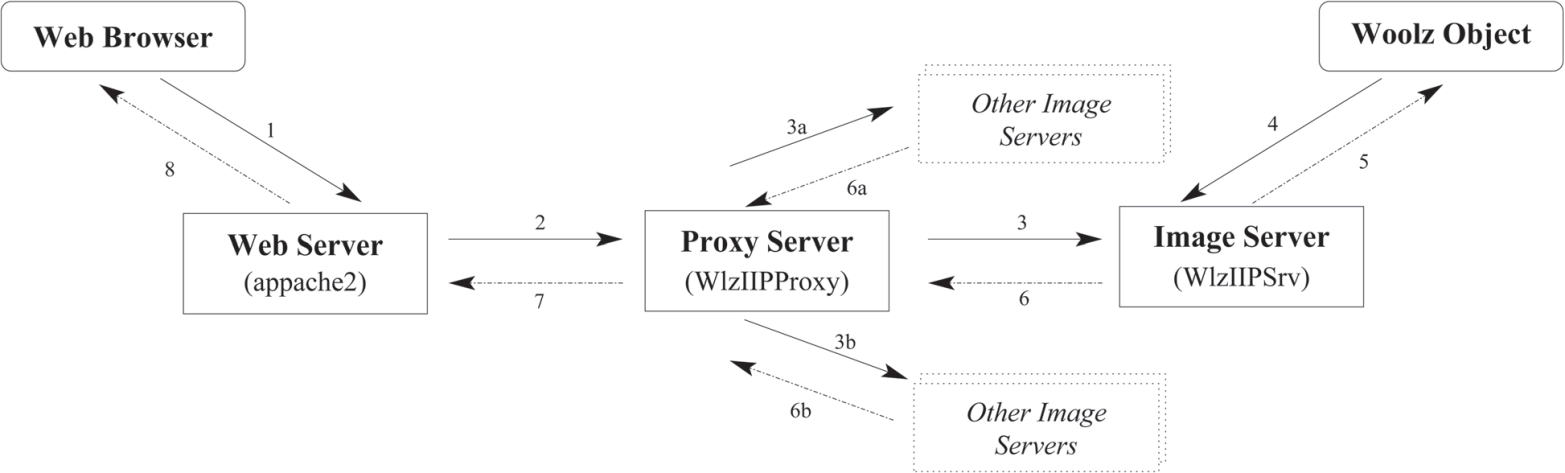
**Architecture of IIP3D server using a proxy server.** The web server passes the user requests to the proxy, which forwards them to individual IIP servers. These servers have direct access to the Woolz Object and return the requested data. The numbered lines show the order of the requests (continuous lines) and the replies (dotted lines).

A multi-IIP3D server architecture is shown in Figure 4. IIP3DProxy is an independent program running on the proxy server. The web server (e.g. Apache 2) forwards it the FCGI request on a configurable IP port, then the HTML request string is checked by IIP3DProxy and if the definition string of any remote IIP3D server is a substring of the request parameters then this query is passed over to the matching server. If no correspondence was found then the request is passed to the default server.

## Results and discussion

### IIP3D server

#### Tiled image

**Figure 5:**
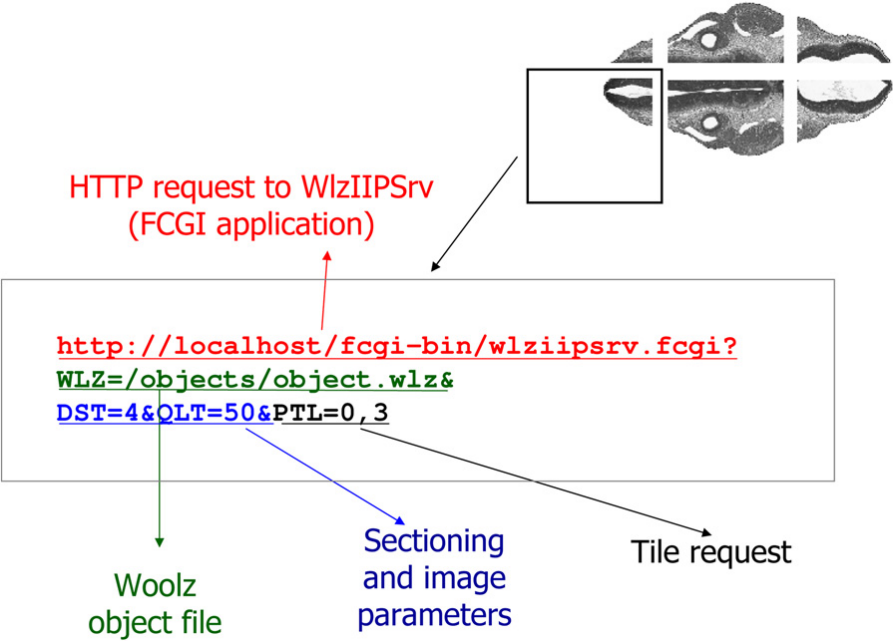
**An section image consisting of 2 × 3 tiles.** The tiles are obtained by 6 requests that are identical except the required tile number of the command.

An example section query that consists of six tiles is shown in Figure 5. The requests, each returning a PNG tile, differ only in the PTL command with tile number sweeping 0 to 6, while all have in common a FCGI application, Woolz object definition () and sectioning image parameters (, , , and ).

#### Overlays with **SEL**

**Figure 6:**
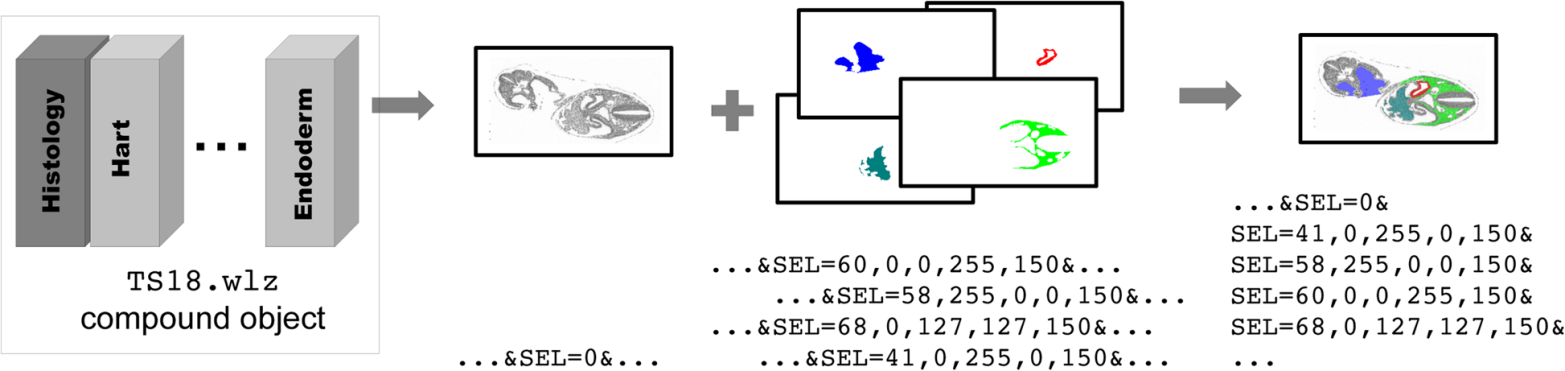
**Multi channel request with*SEL***. The compound Woolz object consists of several channels with multiple domains, each of which is sectioned individually. They may be queried separately or combined into a single layer in sequential order of the command. The colour and transparency values for each layer are selected independently.

The IIP3D server delivers tiled views of images and any given application can request tiles from multiple image volumes to be co-displayed as a series of layers with transparency provided by the browser display capabilities. We have implemented a novel strategy using the IIP3D command which can be used to request graphical overlays stored in an indexed object. This process is depicted in Figure 6 and overlay tiles with anatomy regions defined either individually or collectively can be retrieved and displayed as independent single- or multi-layer, overlay images. The overlay request includes the colour and opacity for requested region. A critical property of the Woolz indexed object is that the domains represented are not limited by the number of bits used for the indexed image and and the regions can overlap i.e. they are not spatially exclusive. the Woolz IIP3D server provides the domain overlay as a single tiled image computed on-the-fly from the Woolz object.

#### Queries

The IIP3D protocol includes extension of the query commands to provide information for image layout and user feedback. These include the size (in pixels) of a given section image, the size of a tile, the voxel size, the object volume and bounding box and the grey or colour value at a given pixel location within the section image. For example, the voxel grey value query with respect to the 3D location (190,200,190) is http://localhost/fcgi-bin/wlziipsrv.fcgi?∖WLZ=/objects/TS18.wlz∖PAB=190,200,190∖OBJ=Wlz-grey-value which results in the reply Wlz-grey-value:73

This query can also be made with respect to the 2D location within a given tile.

#### Performance evaluation

Husz *et al*[20] demonstrated the performance benefit that is realised with a tiled representation with a frame rate of at least 22 fps on a standard WAN and 7 fps to a domestic broadband. However, evaluation under heavy load or simulated user interaction had not been studied. Here we present the results of testing a single IIP3D server with up to sixty simultaneous users. The test consisted of repeatedly running the recorded browsing pattern of a biologist user viewing a cropped Visible Human data set (full colour, 1710x1050x1866 voxels) with a data volume of 12.5 GB. The browsing pattern included tile requests ( ), down-scaled full section request ( ) and object queries ( ), Table 3. The total requested data over 273 seconds of interactive browsing is 3.5 MB, the tile size was 256 × 256. In the following we test two scenarios, first we respect the timing of server requests and second they are ignored which results in a more severe test of the server response. In addition we also test the client requests with and without a random perturbation of the view parameters. This is to ensure all requests are unique and we test the server response without any benefit of caching.

**Table 3:** Test data composition

	JTL	CVT	OBJ
Number	532	104	35
Size[bytes]	3,180,843	334,632	1,261

A series of experiments were performed with a single IIP3D server and 1 to 60 clients. The server and client computers were all equipped with dual 3GHz Intel Xeon 5160 CPUs, 7200 R.P.M. SATA disks and 32GB RAM and are directly connected via a dedicated 1 Gb Ethernet switch. There was no other activity on the server, on the clients or on the network other then operating system management tasks.

For testing, we used standalone C++ executables started simultaneously on the 11 client nodes. For test cases with more clients than available client-nodes, multiple concurrent instances were started on the same client node.

The timestamp of each request was recorded along with all other IIP3D request parameters. This allows replication of user browsing behaviour. The object queried was already prefetched from the disk into the server main memory and the complete set of requests were repeated as whole series and timings captured. The first test set takes 4.88s, while the consecutive requires only 0.77s, suggesting the effect of the local tile cache.

**Figure 7:**
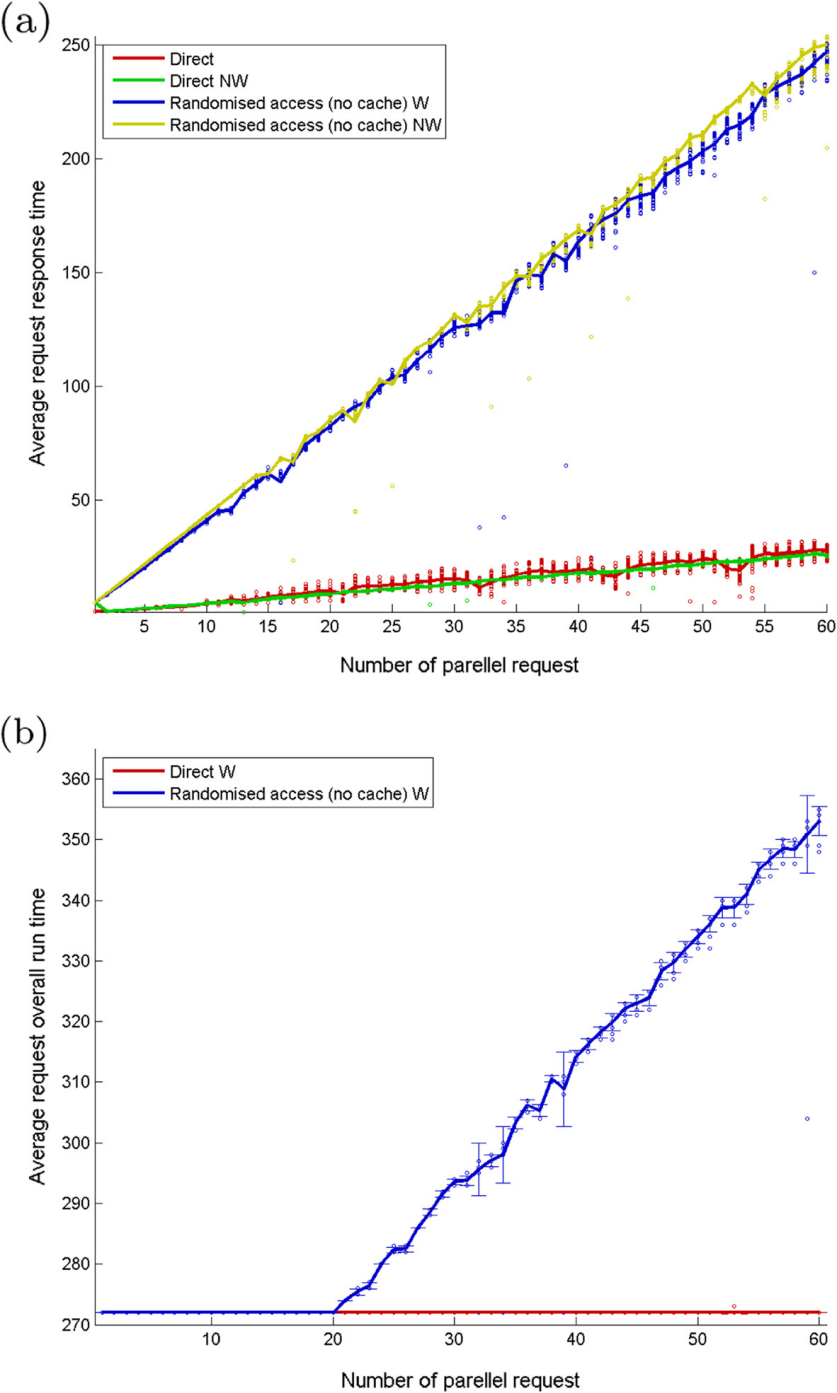
**Performance evaluation for four test cases: the direct sets replicate browsing pattern manually performed by a user over 273 seconds; the randomised requests for each client add a random [0.0; 1.0] value to the requested yaw angle, therefore it forces recomputing the sections and does not use cache.** Both direct and randomised have a wait (W) and no wait (NW) version, which either respect the timing of the manual requests (e.g. waits between consecutive requests) or it does requests tile one after the other. **a**) Average request time per number of concurrent clients, where dots show the individual request times for each client and **b**) Average running time for a test set on clients.

Four different scenarios were evaluated with the results shown in Figure 7. First, we request in parallel the same data on a number of 1 to 60 clients. The average response time increased from 0.77s to 28.80s. The time normalised to the number of clients, shows a constant time behaviour ( 0*.*4651 ± 0*.*0591s), confirming the linearity in Figure 7a.

The timing requests were ignored in the second test scenario, and requests were started one after the other. The server becomes heavily loaded by the simultaneous 671 requests. However, the result graph shows again a linear behaviour, with 0*.*5064 ± 0*.*5641*s* per client response time.With its tile caching, the server avoids re-computation of tiles. Therefore, to force different section generation, we have have altered the original requests by adding a random value to the sectioning plane angle (). Again, Figure 7, there is a linear increase of the processing time with the number of requesters. The average per client time is 4*.*0823 ± 0*.*1183*s* respectively 4*.*2335 ± 0*.*1128*s*. These times are comparable with the initial 4.88s tile cache initialisation for our first test. Key times of these results are summarised in Table 4.

**Table 4:** Summary timing results (in seconds) for different access scenarios described in the text and for selected client numbers

Test	1 client	20 clients	60 clients	normalised
Average response with cache	0.77	9.1	28.80	0.46 ± 0.06
Average response no cache	4.1	82.6	246	4.23 ± 0.11

The normalised figure is the average per client.

It is interesting to remark in Figure 7b that the running time of the timed test sets recorded over 273 seconds, it is respected for the cached scenario, while for the non-cached scenario up to 20 clients managed to fit in the time user needed to browse through the object. This suggests that up to 20 clients may be served without major performance degradation, however as is shown above the display delay increases.

Client executables were started remotely from a test script. We had no control on the exact timing of these processes neither how the request are prioritised by the apache web server, therefore occasionally “lucky” clients occur with lower execution time then the average. Note the client executable did not use any local caching therefore the benefit of the browser cache is not included with these measurements which are therefore a lower bound on the performance experienced by the user.

### IIP3D viewer

IIP3D is configurable for the application in use. We present the basic 3D browsing functionality, followed by the support of handling overlaid 3D image data and large databases. We exemplify these functionality on the male VisibleHuman dataset [24], adult mouse muti-modality Waxholm data [25], HUDSEN developing human brain atlas [26], fly brain atlas [27] and the eMouseAtlas atlas models [28].

#### 3D browsing and multi-channel data

**Figure 8:**
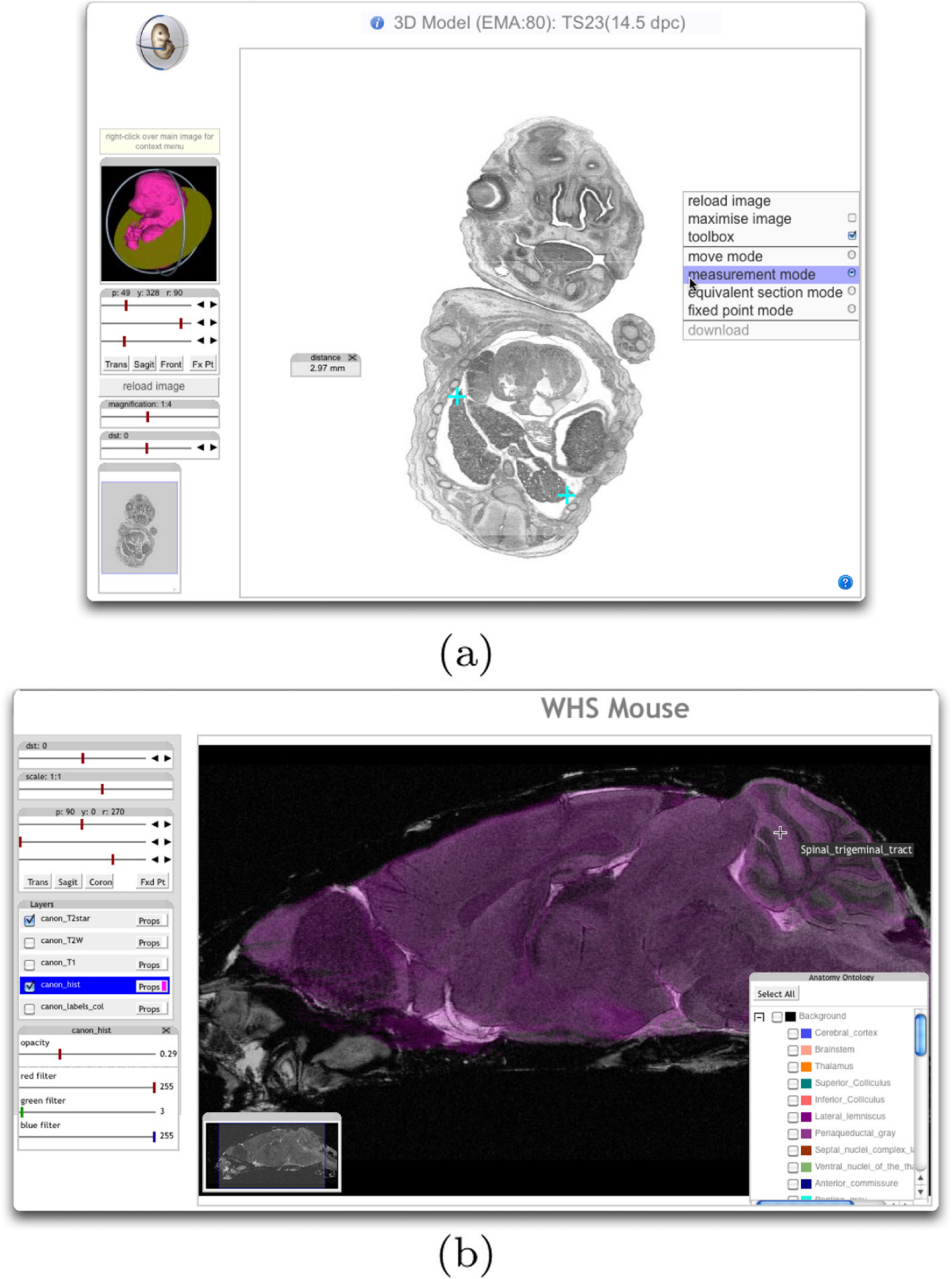
**Browsing 3D data using the IIP3D web-browser interface.** (**a**) view of the eMouseAtlas.org E14.5 mouse embryo model. The left-hand side panel provides slider controls to adjust the sectioning orientation which is indicated by the 3D rendering. In the main area the virtual section is displayed with the context menu which indicates the modes available. In this view the measurement mode has been selected and the two cyan crosses indicate the transverse distance across this view of the liver. (**b**) Waxholm space P56 mouse brain. In this view two of the possible 5 modalities have been selected and displayed as two layers. The T2* MR image in grey is overlaid with the histology reconstruction shown as a magenta overlay set to be partially transparent.

Figure 8 shows examples of browsing histological data. Parallel sections from objects are cut with the distance section tool and sectioning angles may be changed setting angle values as in Figure 8a or by choosing a standard view (in this case sagittal) as in (Figure 8b. The magnification tool allows zooming to details of the object (Figure 8a,b), with the visible region shown in the locator window, this can be used to move the view. Tools are attached to a toolbar or may float on the page and are also collapsible. In Figure 8a we show an arbitrary section through the E14.5 (Theiler stage 23, http://emouseatlas.org/eAtlasViewer_ema/application/ema/wlz/EMA80.php) mouse embryo and illustrate the measurement mode option. The IIP3D protocol provides a query capability for a 2D position in a given tile. In this case the server returns the corresponding original 3D location for a given point to enable a calculation of distance between the two marked points. In Figure 8b we show the multi-channel display capability of the IIP3D viewer. For this example we have used the set of five 3D reconstructed images that are part of the Waxholm space atlas standard. These are three MR images (T2*, T2 weighted, T1), stained histology and a labelled tissue segmentation. Applications currently available are not able to simultaneously display sections form all five volumes primarily because of memory constraints of a typical desktop. Here the sections can be displayed in a web-browser and are presented as a *layer* model as found in graphical packages such as Adobe Illustrator and the open-source GIMP. The view selected shows the T2* image overlaid with a colour filtered section through the histology reconstruction. Colour filtering is part of the IIP3D protocol.

#### Large volumetric data

**Figure 9:**
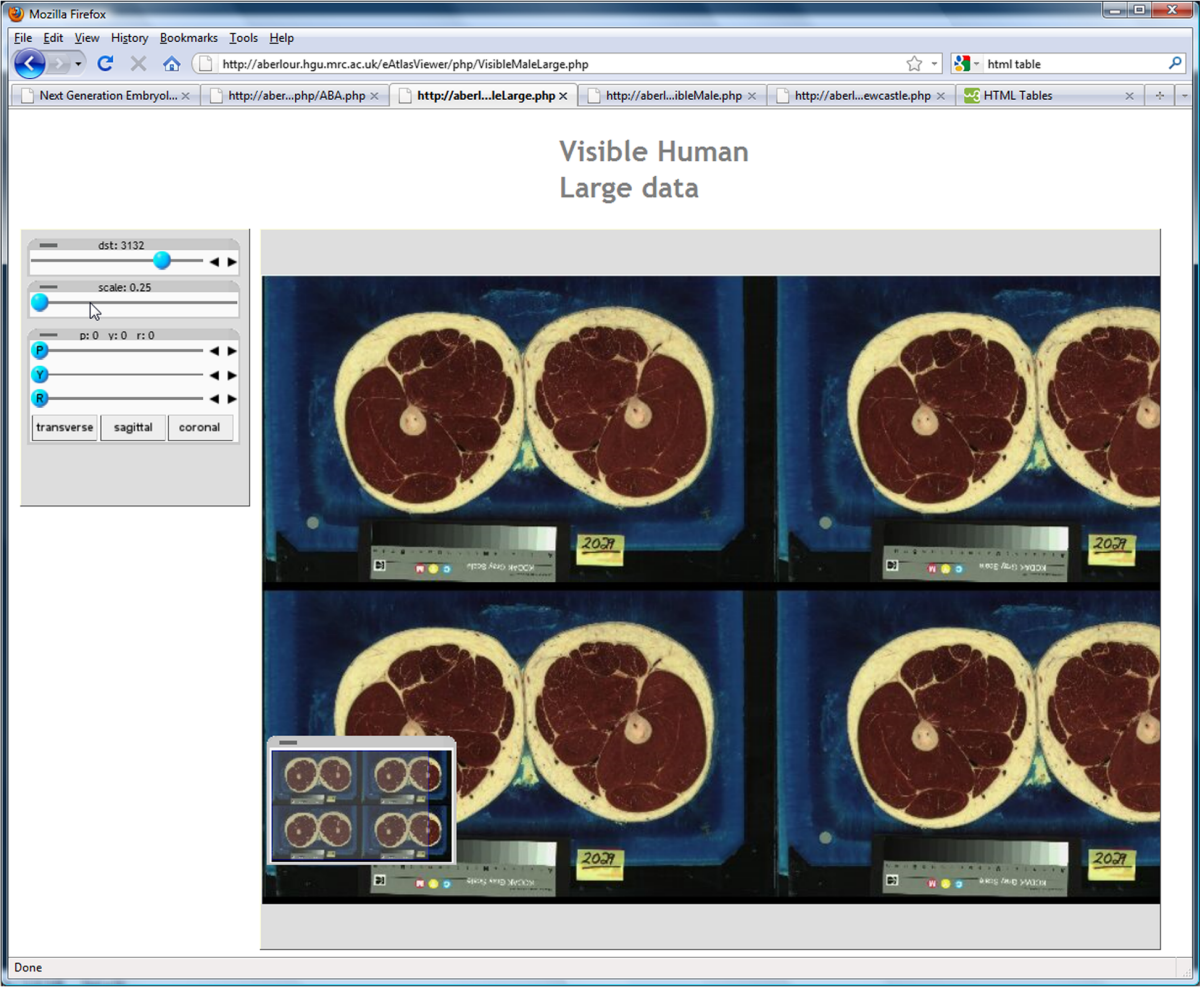
**Example of a large image object.** Visible Male 140GB dataset, with tools anchored to the left area and viewing regions at the right. This figure also illustrates that the IIP3D server can provide section views through colour volumetric images.

Large objects stored and visualised at high resolution may be read and served by the IIP3D server. These use the memory mapped data-structures that avoid the requirement to read the full dataset into main memory which for the larger volumes would not be feasible. In addition for archive data of many 1000s of smaller 3D volumes it enables fast access to any image where again it is not feasible to maintain the image data in memory. We have tested the system for image volumes ranging from 5 to 138 GB and found that the response was slightly slower but acceptable given that these are section views that can not for realised by *any other system*. In Figure 9 we show a view through a composite image comprising 8 full sets of the visible male 3D volume. This results in a 3D image with 4048 × 2432 × 3732 full colour (rgb *α*) voxels with a data volume of about 138GB. The memory mapped data format also allows us to routinely serve sections views from a multiple 3D image archive which now exceeds 4TB.

#### Annotation overlay

Figure 8b shows concurrent viewing of image layers with control of a colour filter and transparency across each image layer. The SEL command enables fine-grained control of colour and transparency of annotation overlays which can be considered as equivalent to an indexed image. An index defines a region of the 3D image space and the intersection of that 3D region with the current section will return tiles with those pixels given the requested colour and transparency. If two regions overlap in the same region then the colours will be merged. The result is a single layer that can be used to show regional annotations for example anatomical delineations or the presence of gene-expression. Pointing on the grey value brings up the name of the underlying single or multiple domain, if available. This is possible because the IIP3D compound object formalism allows annotated regions to overlap and there is no constraint in number.

**Figure 10:**
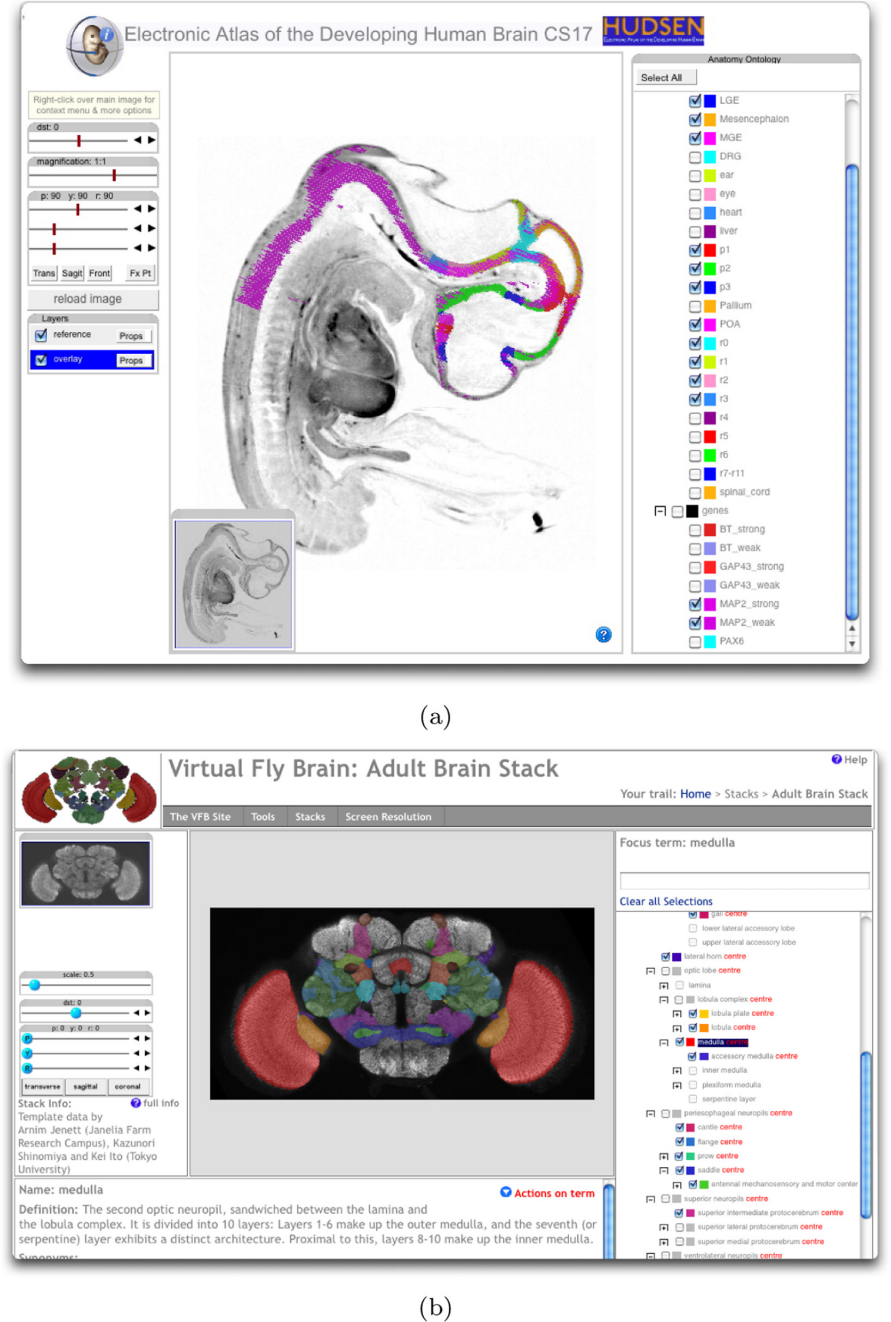
**Layered browsing.** (**a**) shows a sagittal view through the HUDSEN CS17 human embryo with anatomy delineation and in situ gene-expression overlay. Pausing the cursor over a painted region will display the name of the underlying region and mouse double-click will result in selection of the matching term in the ontology display tool. (**b**) shows the virtual flybrain interface which has been further developed to enable query of anatomy term definitions and associated gene-expression and connectivity databases.

Figure 10 illustrates the use of IIP3D to show anatomy overlay regions providing full control to the user to select which terms should be visible and the corresponding colours and transparencies. We show two examples. Figure 10a is a view through the HUDSEN CS17 embryo (http://hudsen.org) with selected anatomical regions displayed plus the expression domain of the gene MAP2. Figure 10b is a screen-shot of the new Virtual FlyBrain interface (http://virtualﬂybrain.org), which also delivers access to a database of brain-structure connectivity and gene-expression via context menu associated with the anatomy tree.

## Conclusions

We have extended a protocol standard for tile-based viewing of 2D image data to include 3D volumetric data to enable virtual re-sectioning at arbitrary orientations through the volume. The protocol is also extended to handle index-image data giving control to the user of colour and transparency on each individual labelled region. On the basis of this protocol we have implemented an IIP3D server to deliver tiles for any requested section and a Javascript/Ajax viewer which provides a user-interface that will run within a web-browser. Performance tests on the server show that it can handle up to 60 simultaneous users without significant loss of performance as experienced by a typical user. The server can be scaled using a series of proxies to provide the required performance for higher access rates as required. The server code is implemented on a standard Linux-based machine and does not require special a HPC facility.

We have tested this system in the context of atlas-based biological data however it is clearly applicable to a wide range of image data for which arbitrary re-sectioning is a useful visualisation. This visualisation is typically the core requirement in basic science but also medicine where views through the data are the primary views needed by experts who want to see “the real data”. It is complementary to recent development of in HTML5 in particular WebGL which is exemplified by the Google “Body Browser” demonstrator (http://bodybrowser.googlelabs.com). We plan to use IIP3D in a future WebGL based application to provide cut section image textures.

For archives of volumetric data or single very-large image volumes this server development is the only plausible mechanism to deliver views to users who do not have the required bandwidth, local storage capability or the compute capability to be able to download and view the data. Here we realise the benefit of low-bandwidth tile-based access to image data coupled with very efficient image-processing to calculate the virtual section tiles. This technology is open-source and will run on any unix-based web-server.

The IIP3D server allows access to independent software either as a standalone application or a more complex Java or Adobe Flash viewer. However, for flexibility and interoperability we provide a JavaScript API and viewer which runs in most up-to-date Internet Browser software, e.g. Internet Explorer, Safari, Firefox and Chrome but with some variation on behaviour arising from different ways in which the browsers have implemented standards (see the eMouseAtlas tested systems^1^).

Through the IIP3D API the 3D viewer can be integrated into complex applications such as Next Generation Embryology^2^ - a 3D spatio-temporal framework that augments IIP3D data in conjunction with a repository to deliver research and educational material. Users may add supplementary augmentation (points or region) to the 3D volume, together with texts, videos, external links.

The compound representation of multiple images (i.e. grey level, colour or segmented domains) and providing them as individual or composed images allows compact representation of related data, and offers a novel 3D visualisation of overlays. Also, it has power of representing overlapping domains that standard index-image representations can not provide. We have shown that the performance of our implementation of IIP3D scales well with the number of parallel requests. Grey level or colour data is successfully augmented with domain or gene expression data. Controls, such as sliders, layer selection tool or locator resemble functionality of know tools from desktop applications. These tools and the associated tiling code are all available as open-source.

Future developments of this software include grey-level transforms such as range slicing for 16bit medical data, extension to vector and scalar data and the introduction of a standard glyph library for location and directional marking. In the context of the client interface we will address usability by undertaking a user-evaluation study.

## Availability and requirements

· **Project name:** Woolz IIP

· **Project home page:**http://www.emouseatlas.org/emap/analysis_tools_resources/software/wlziip.html

· **Operating system(s):** sever: Linux; client: platform independent

· **Programming language:** server: C and C++; client: JavaScript

· **Other requirements:** server: Woolz X.X, FastCGI library and web server with FastCGI enabled; client: Internet browser must support JavaScript and display images

· **License:** GNU GPL

· **Any restrictions to use by non-academics:** according to GNU GPL

## Endnotes

^a^http://www.emouseatlas.org/emap/help/compatibility.html^b^http://research.nesc.ac.uk/node/400

## Abbreviations

IIP: Internet Imaging Protocol; IIP3D: 3D extended Internet Imaging Protocol; FCGI/FastCGI: Fast Common Gateway Interface.

## Competing interests

The authors declare that they have no competing interests.

## Author’s contributions

ZH designed and extended the original IIP protocol, coded the IIP3D server, suggested the existing compound Woolz object representation for domain data and wrote the first draft of this paper. BH and RB develop the Woolz image processing library, RB developed the Woolz sectioning algorithm and BH added support for memory mapped objects. NB integrated features of existing browser to provide a unified application programmer interface (API). NM added support of tree selection of anatomical hierarchy for overlaid images and provide input for viewer API standardisation. RB formulated the underlying ideas for using the IIP server for section data and managed the design and software development. All authors read and approved the final manuscript.
